# Decellularized allogeneic intervertebral disc: natural biomaterials for regenerating disc degeneration

**DOI:** 10.18632/oncotarget.7735

**Published:** 2016-02-25

**Authors:** Xianfeng Lin, Xiangqian Fang, Qiang Wang, Zhijun Hu, Kai Chen, Zhi Shan, Shuai Chen, Jiying Wang, Jian Mo, Jianjun Ma, Wenbing Xu, An Qin, Shunwu Fan

**Affiliations:** ^1^ Department of Orthopaedic Surgery, Sir Run Run Shaw Hospital, Medical College of Zhejiang University, Hangzhou, China; ^2^ Institute of Micro-Invasive Surgery of Zhejiang University, Hangzhou, China; ^3^ Department of Orthopaedic Surgery, the First Affiliated Hospital of Wenzhou Medical College, Wenzhou, China; ^4^ Department of Orthopedics, Shanghai Key Laboratory of Orthopedic Implant, Shanghai Ninth People's Hospital, Shanghai Jiaotong University School of Medicine, Shanghai, China

**Keywords:** intervertebral disc, decellularization, extracellular matrix, disc degeneration, mesenchymal stem cell, Pathology Section

## Abstract

Intervertebral disc degeneration is associated with back pain and disc herniation. This study established a modified protocol for intervertebral disc (IVD) decellularization and prepared its extracellular matrix (ECM). By culturing mesenchymal stem cells (MSCs)(3, 7, 14 and 21 days) and human degenerative IVD cells (7 days) in the ECM, implanting it subcutaneously in rabbit and injecting ECM microparticles into degenerative disc, the biological safety and efficacy of decellularized IVD was evaluated both *in vitro* and *in vivo*. Here, we demonstrated that cellular components can be removed completely after decellularization and maximally retain the structure and biomechanics of native IVD. We revealed that allogeneic ECM did not evoke any apparent inflammatory reaction *in vivo* and no cytotoxicity was found *in vitro*. Moreover, IVD ECM can induce differentiation of MSCs into IVD-like cells *in vitro*. Furthermore, allogeneic ECM microparticles are effective on the treatment of rabbit disc degeneration *in vivo*. In conclusion, our study developed an optimized method for IVD decellularization and we proved decellularized IVD is safe and effective for the treatment of degenerated disc diseases.

## INTRODUCTION

Intervertebral disc degeneration (IDD) is associated with back pain and disc herniation [1]. It is estimated that 84% of the people have experienced low back pain (LBP) during their lifetime, and the costs related to low back disorders exceed $100 billion per year in the USA [2, 3]. IDD can usually lead to multiple spinal disorders such as disc herniation, spondylolisthesis and spinal stenosis [4]. Although there are many nonoperative management strategies to relieve the symptoms temporarily, discectomy and fusion are still the principal measure of treatment [5, 6]. However, spinal fusion changes the biomechanical properties of the spine and thus may accelerate adjacent segment degeneration [7].

Recent advances in synthetic materials and biological techniques have been adopted to simulate the architecture and function of the intervertebral disc (IVD) [8-16]. Since native interevertebral disc has a complex three-dimensional architecture and unique biological characteristics, novel strategies that are more effective and have fewer long-term side effects are needed.

It appears that regenerating degenerative organs could theoretically be one of the choices for overcoming the aforementioned hurdles. Decellularization is an attractive technique for preparing the specific organ or tissue extracellular matrix (ECM) scaffold. ECM could serve to regenerate organs and tissues [17]. Researchers use physical (e.g., shocks and freeze/thaw cycles), enzymatic (e.g., DNAase and trypsin) and chemical (e.g., Triton X-100 and sodium dodecyl sulfate (SDS)) protocols to decellularize organ or tissue of interest to acquire the organ-specific ECM [18]. For example, repeating freeze/thaw cycles can disrupt cellular membranes and cause cell lysis, and chemical protocols can relatively mild effects upon tissue structure and solubilize both cytoplasmic and nuclear cellular membranes. Through removing allogenic or xenogenic cellular components, one could theoretically use these ECM to produce a minimally immunogenic scaffold with the proper biomechanical and biological properties. Indeed, several studies have tried to decellularization methods for regenerate IVD. Simionescu and Xu et al have decellularized the individual native-derived nucleus pulposus (NP) or annulus fibrous (AF) tissue [19-21]. Cheung et al [22] have attempted to decellularize a complete IVD, while only 70% of the endogenous cells were removed and complete decellularization was not achieved. Chan et al choose collagen type I as the main structure to obtain a cell-derived decellularized NP [23]. However, there could be some problems because the major components of native NP ECM were collagen type II and glycosaminoglycans (GAGs). Here in our study, we try to decellularize the whole intervertebral disc completely and use this native ECM scaffold to treat disc degeneration.

## RESULTS

### Optimized decellularization method preserved ECM components of IVD

**Figure 1:**
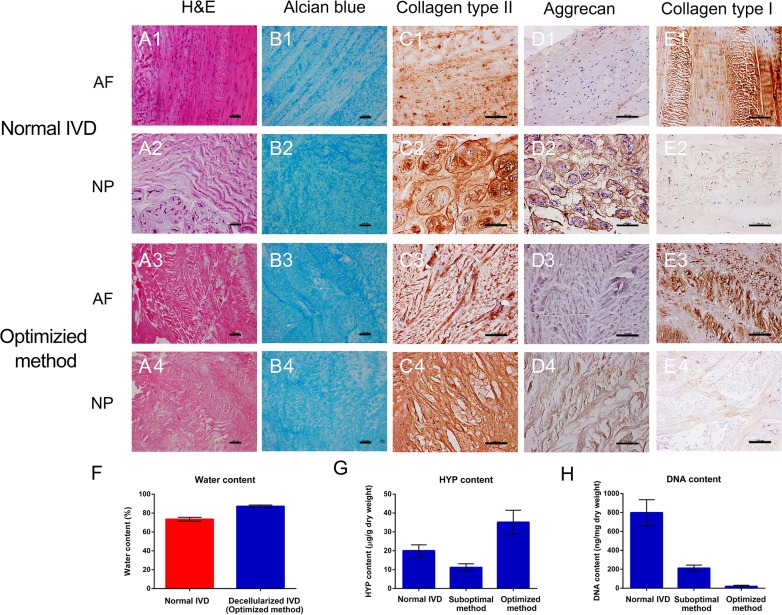
Optimized decellularization method preserved ECM components H&E, Alcian blue, collagen type II, AGN and collagen type I staining of normal **A1.**-2, **B1.**-2, **C1.**-2, **D1.**-2, **E1.**-2 and optimized method **A3.**-4, **B3.**-4, **C3.**-4, **D3.**-4, **E3.**-4. The content of DNA **F.**, HYP **G.** and water **H.** revealed the excellent biological characteristics of optimized method group (*n* = 5 per study group). Optimized method includes 2% Triton X-100 for 24 h, 1% SDS for 24 h and 200 U/ml DNAase for 12 h. Abbreviations: IVD = intervertebral disc, AF = annulus fibrous, NP = nucleus pulposus, HYP = hydroxyproline. A-E: Scale bar 100μm.

Different decellularization methods were tried before an optimal method was chosen for further study. After the whole IVD was decellularized using the optimal decellularization method, hematoxylin and eosin (H&E) staining was performed to check the overall effect of the decellularization process on the removal of the cellular components. As shown in Figure 1A, 1H & 1E staining of the decellularized IVD tissues showed pink eosinophilic staining that is typical of collagen, which was comparable with normal IVD. In contrast, no basophilic staining (indicative of cellular nuclear material) was detected in the decellularized tissues. Alcian blue staining (Figure 1B) revealed that the content of the GAGs is not reduced after decellularization. Moreover, comparable immunohistochemical staining revealed that the principal components of the IVD ECM, including collagen type II, aggrecan (AGN) and collagen type I, were remained well in the optimized method group (Figure 1C, 1D and 1E). After decellularization process, the collagen type II and AGN also abound in NP and collagen type I still exists largely in AF. Interestingly the contents of hydroxyproline (HYP) and water were even increased in optimized method group (20.05 ± 3.09 μg/g *vs*. 35.15 ± 6.35 μg/g, *P* = 0.000; 73.52 ± 2.03% *vs*. 87.22 ± 1.34%, *P* = 0.000) (Figure 1F and 1G). Furthermore, the degree of DNA removal in optimized method could reach up to 97.59 ± 1.50 % (*P* = 0.000) (Figure 1H). The destructive effect of suboptimal method on ECM components was evaluated and shown in Supplemental Figure 3. Therefore, optimized method using 2% Triton X-100, 1% SDS and 200 U/ml DNAase was chosen as the final decellularization protocol for further study.

### Optimized decellularization method preserved ECM structure and mechanical properties

**Figure 2:**
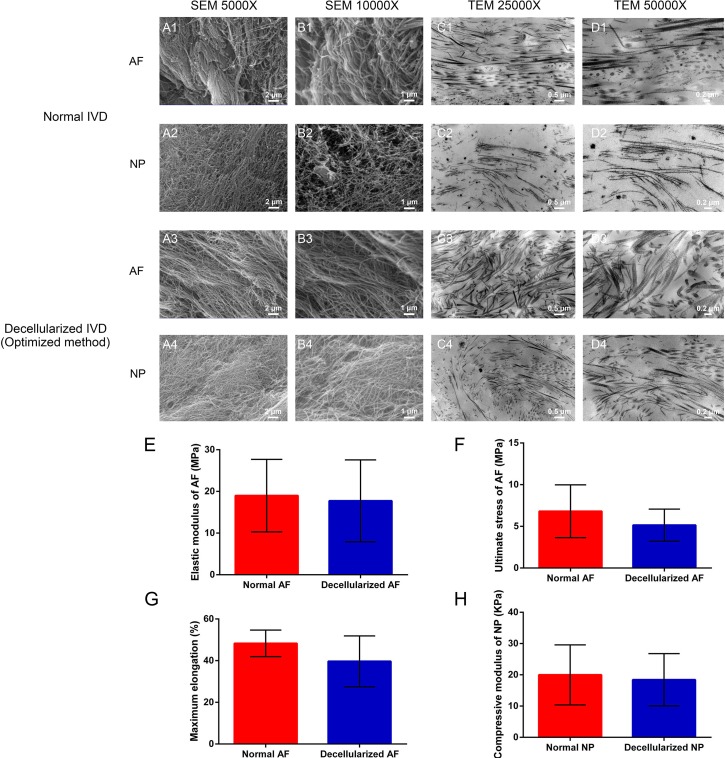
Optimized decellularization method preserved ECM structure and mechanical properties Ultrastructure analysis reaveled that the collagen fibril meshwork in optimized method group **A3.**-4, **B3.**-4, **C3.**-4, **D3.**-4 is preserved and is similar to normal IVD **A1.**-2, **B1.**-2, **C1.**-2, **D1.**-2. The elastic modulus **E.**, ultimate stress **F.** and maximum elongation **G.** indicate the mechanical change in AF after decellularization. The compressive modulus **H.** of normal NP and decellularized NP were measured at a 1 Hz loading frequency (*n* = 6 per study group). Abbreviations: IVD = intervertebral disc, AF = annulus fibrous, NP = nucleus pulposus.

In addition completely removal of cells and the preservation of extracellular components, the preservation of ECM structure and mechanical property were also investigated. Scanning electron micrography (SEM) and transmission electron micrography (TEM) were applied to show the ultrastructural characteristics of ECM scaffold (Figure 2). SEM showed a well-organized parallel-aligned collagen fiber network in normal AF (Figure 2 A1 and 2 B1). In the decellularization group, the collagen fibers of the decellularized AF appeared to be more regular, and they also have a tiny and homogeneous space between the fibers compared with normal AF (Figure 2 A3 and 2 B3). The collagen of normal NP was intertwined and disorganized, which was similar to a complex meshwork that has fibers intercrossing between layers (Figure 2 A2 and 2 B2). The pores between the collagen fibers vary widely in normal NP. A similar pattern was found in the decellularized NP. The cross-cutting and mesh structure was well preserved after decellularization (Figure 2 A4 and 2 B4). The images of TEM further confirmed that the integration and continuity of the microstructure of IVD were well preserved after decellularization (Figure 2C and 2D). Together, these data suggested our decellularization methods well preserved the contents of ECM. In addition, the mechanical properties of decellularized IVD were retained well compared with normal IVD (Figure 2). The elastic modulus of annulus fibrous in decellularization group was similar to the than normal AF (17.74 ± 9.83 *vs*. 18.98 ± 8.70 MPa, *P* = 0.821). The ultimate stress (UTS) and maximum elongation were also not obviously affected after decellularization (Figure 2F and 2G, *P* = 0.323 and 0.202, respectively). In addition, the compressive modulus of the decellularized NP was comparable to that of the normal NP (18.41 ± 8.38 *vs*. 20.00 ± 9.59 kPa, *P* = 0.801) (Figure 2H). Collectively, the optimal decellularization methods preserved both ultrastructure and mechanical properties of the whole IVD.

### Decellularized allogenic IVD induced minimal immunological reaction *in vivo*

**Figure 3:**
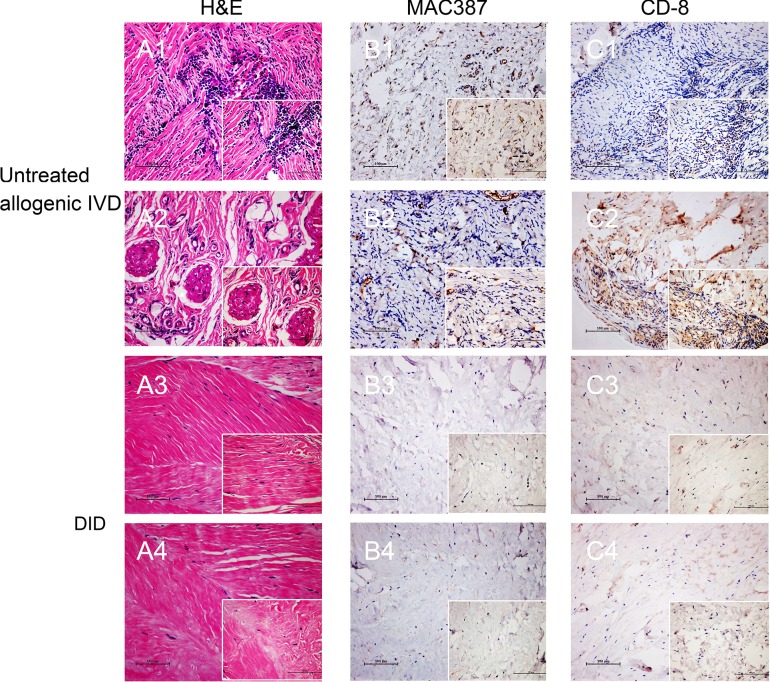
Decellularized allogenic IVD induced minimal immunological reaction *in vivo* H&E, MAC387 and CD-8 staining of untreated allogenic IVD **A1.**-2, **B1.**-2 and **C1.**-2 and decellularized allogenic IVD **A3.**-4, **B3.**-4 and **C3.**-4 IVD were performed after 1 month of subdermal implantation in rabbits. * Blood vessels, ▲ Foreign body granulomatous inflammation, → positive signal of MAC386 or CD-8. Abbreviations: IVD = intervertebral disc, DID = decellularized intervertebral disc.

As immunological response is critical for allogenic implant, the immunological response was first evaluated *in vivo*. All the animals survived, and no complications were observed during one-month implantation period. The histological evaluation indicated a high degree of host cell penetration into the native IVD, whereas only a few host cells were scattered in the decellularized IVD (Figure 3A). Neutrophils were observed extensively scattered around the grafts, which suggested a severe inflammatory response (Figure 3 A1). In addition, new blood vessels and classic foreign body granulomatous inflammation were found in the untreated allogenic IVD (Figure 3 A2). In contrast to the above inflammatory reaction, fewer inflammatory cells were observed, with only a small amount of fibroblasts dotted in the decellularization group (Figure 3 A3 and 3 A4). Immunostaining of the macrophages and CD8+ T-cells was used to determine the degree of cell-mediated immunoreaction. A large number of infiltrating CD8+ T-cells and macrophage cells were found in untreated allogenic IVD group (Figure 3 B1-2 and 3 C1-2). However, the percentage of positive cells of was fewer in decellularization group (Figure 3 B3-4 and 3 C3-4). Besides, the *in vitro* cytocompatibility of decellularized IVD was tested by cell counting kit-8 (CCK-8) and Live-Dead cell staining (Supplemental Figure 4) suggesting decellularized IVD has good cytocompatibility *in vitro*. Taken together, these results suggested that the decellularization process avoided a cell-mediated immunoreactions to the grafts.

### Decellularized IVD ECM supports mesenchymal stem cell (MSC) proliferation *in vitro*

**Figure 4:**
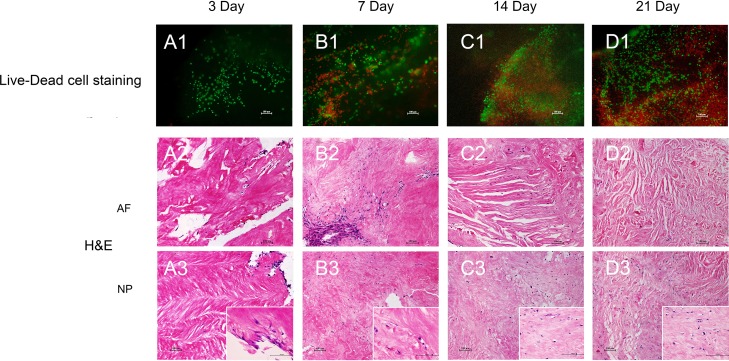
Decellularized ECM supports MSC proliferation *in vitro* Live-Dead cell **A1.**-**D1.** and **H.**&**E. A2.**-**D2.** and **A3.**-**D3.** staining of the MSC proliferation process. Abbreviations: AF = annulus fibrous, NP = nucleus pulposus.

For potential tissue regeneration application, the effect of decellularized IVD ECM on MSC was first evaluated *in vitro*, and before that, the native disc cells were confirmed completely removed after decellularization process (see above). Live-Dead cell staining indicated long-term viability of the MSCs after being seeded for 3, 7, 14 and 21 days (Figure 4). The live cells (green) showed that the majority of the seeded cells migrated into the inside of decellularized disc from day 3 to day 21 (Figure 4 A1-4 D1). The cells were predominantly found on the surface of the decellularized disc, with a small number of cells migrating into the inner region on day 3 (Figure 4 A1). As time progressed, more and more seeded cells proliferated and migrated into the decellularized disc (Figure 4 B1 and 4 C1). Even more intriguing, the seeded cells were distributed evenly throughout the decellularized disc on day 21 (Figure 4 D1). Furthermore, H&E staining showed similar migration pattern. It is noticeable that the seeded cells are elongated and lined up along the collagen fibers in AF (Figure 4 C2). More interestingly, the cell morphology changed from fusiform to round or oval at the site of the NP (Figure 4 C3 and 4 D2-3). 4, 6-diamidino-2-phenylindole (DAPI) staining was used to further confirm the MSCs proliferation in ECM (Supplemental Figure 5). Additionally, cell metabolic activity and proliferation of MSCs were also assessed by the CCK-8 and DNA content assay, which were in agreement with the above results (Supplemental Figure 5).

### Decellularzied IVD ECM induced IVD-like cell differentiation *in vitro*

**Figure 5:**
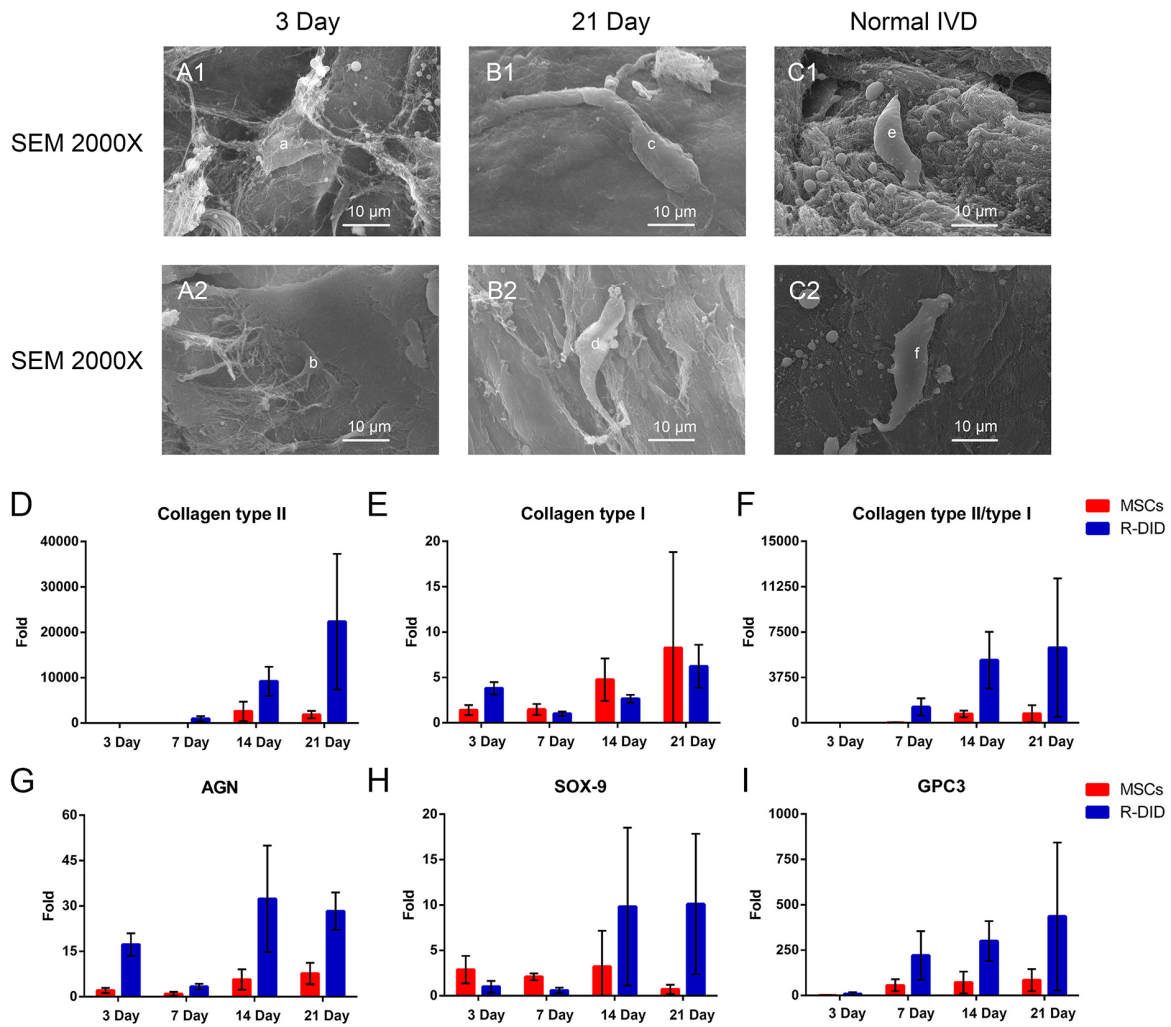
Decellularzied IVD ECM induced IVD-like cell differentiation *in vitro* The dynamic changes in the cell morphologies **A1.**-2 and **B1.**-2 were observed, and normal IVD **C1.** and **C2.** was used as the reference. “a” and “b”: Cells seeded into decellularzied IVD ECM on day 3; “c” and “d”: Cells seeded into decellularzied IVD ECM on day 21; “e” and “f”: Normal IVD cells. The classic IVD-related gene expression of MSC seeded in ECM by RT-PCR **D.**-**I.**. Gene expression data are normalized to the average number. MSCs: Cells cultured in standard DMEM-HG medium (*n* = 8 per study group per time point). Abbreviations: IVD = intervertebral disc, R-DID = recellularized decellularization IVD, AGN = aggrecan.

Our histology result revealed morphological changes when MSCs cells are seeded on the decellularized IVD. Therefore, we further evaluated the morphology of these cells using SEM. SEM found IVD-like cell differentiation was induced with MSCs seeded in the decellularized IVD. The dynamic morphology changes were observed in Figure 5. At low magnification (×2000), the thin and flat cell anchored onto the decellularized IVD surface, stretching with lengths that ranged to approximately 10 μm on the three-day (Figure 5 A1-5 A2). After 21 days of culturing, the cell body translated into a column or strip-like shape, and the cell shape was similar to normal intervertebral disc cells (Figure 5 B1-2 and 5 C1-2).

In addition to morphological observations, we compared the expression of IVD-related genes between cells that were seeded in decellularized IVD and non-seeded MSCs. Collagen type II is one of the most important IVD cell markers. The expression of collagen type II progressively increases during the culture period in the decellularization group. The expression pattern is consistently higher in the decellularization group than the control group, with an increase of more than 12-fold in the recellularized group on day 21 (Figure 5D). In contrast, no significant difference was found in the expression of collagen type I (Figure 5E). The expression of SOX-9, a classic IVD cell marker, was fourteen times (*P* < 0.05) greater than in the non-seeded MSCs in later stages (Figure 5H). In agreement with this finding, the expression of the other IVD-cell markers, AGN and glypican 3 (GPC3), were significantly greater at different time points (Figure 5G and 5I). The novel IVD marker genes, forkhead box F1 (FOXF1) and carbonic anhydrase 12 (CA12) also showed large increases (118-fold and 5-fold, respectively, *P* < 0.05) in the decellularization group (Supplemental Figure 6A and 6B). Two-way ANOVA showed that both time (*P* < 0.05) and group (*P* < 0.05) significantly affected the gene expression of AGN, GPC3, CA12 and FOXF1 and collagen type II/type I. Additionally, no significant changes were found in tissue inhibitor of metalloproteinase 1 (TIMP-1) and TIMP-2 expression (Supplemental Figure 6C and 6D). Transforming growth factor β (TGF-β) families and their receptors, especially during the latter stages, were up-regulated in MSCs seeded decellularized IVD (Supplemental Figure 6E-6H). In all, these results suggested decellularized IVD supported IVD-like cell differentiation *in vitro.*

### Decellularzied allogeneic IVD prevented disc degeneration *in vivo*

**Figure 6:**
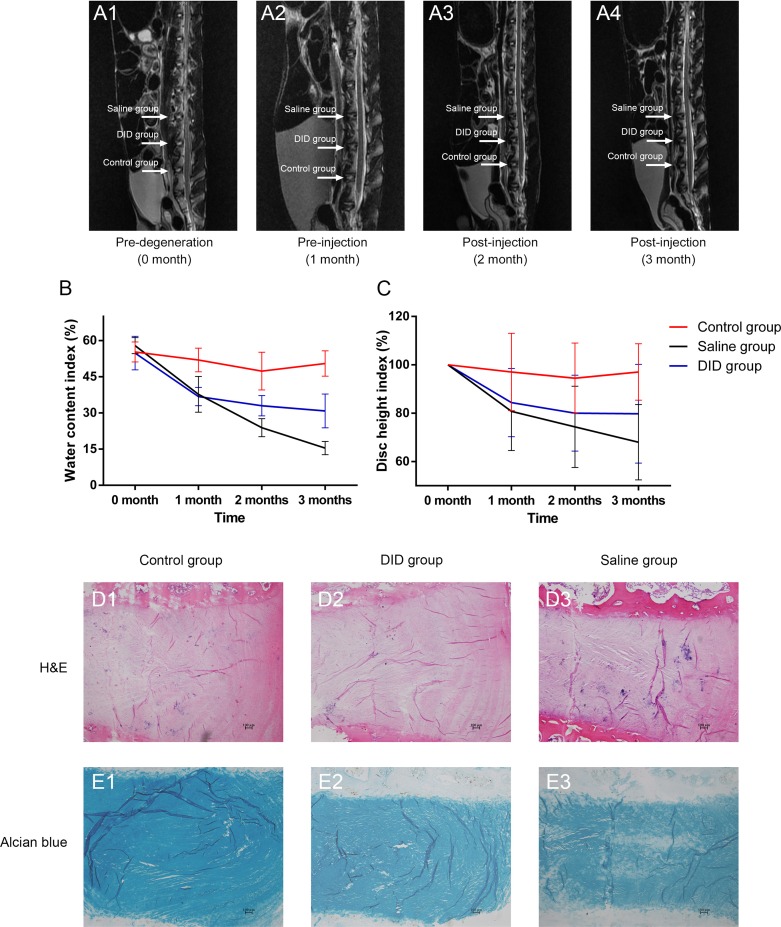
Decellularzied allogeneic IVD prevented disc degeneration *in vivo* Continuous and dynamic changes of IVD magnetic resonance imaging (MRI) on T2-weighted images at different time points **A.**. Water content index **B.** and disc height index **C.** indicated by MRI. H&E and Alcian blue staining of normal (control), DID and saline IVDs at 2 months after injection (*n* = 5 per study group per time point). DID group: allogeneic IVD-derived ECM microparticles. Abbreviations: DID = decellularized IVD.

Given the potential of inducing MSC differentiate into IVD-like cell *in vitro*, we further evaluated the therapeutic effect of decellularized IVD *in vivo*. The effect of decellularzied allogeneic IVD microparticles was used to treat degenerative disc in rabbit. The continuous and dynamic changes of IVD in different groups at 0 month, 1 month, 2 months, and 3 months were demonstrated in Figure 6A. The water content index (Figure 6B) showed that decellularized IVD maintained a higher hydration level compared to the saline group, at both 2 months and 3 months (32.96 ± 4.24% *vs*. 23.90 ± 3.77%, *P* = 0.001 and 30.82 ± 7.00% *vs*. 15.45 ± 2.76%, *P* = 0.005, respectively). Magnetic resonance imaging (MRI) showed that the disc height (Figure 6C) of the decellularized IVD treated group was also slightly better than the saline group, although a significant difference was not observed (*P* = 0.998 and 0.281, respectively). Figure 6B and 6C indicated that the decrease of water content index and disc height was delayed to some extent in the decellularized IVD treated group. Moreover, in the decellularized IVD treated group, the histological morphology of collagen staining by H&E did not show obvious changes compared with the control group. However, in the saline group, the inner layer of the AF lost the concentric lamellar structure with cracks, and the NP was also disorganized (Figure 6D). Alcian blue staining also shows that the obvious loss of GAGs in the saline group. But this loss was not apparent in the decellularized IVD treated group (Figure 6E). For future potential clinical applications, human-derived IDD cells (HDCs) were further seeded to investigate its therapeutic effect. Gene expression profiles of HDCs seeded in the decellularized IVD at day seven were measured. As shown in Supplemental Figure 7, the degeneration-specific markers, collagen type I and collagen type III expression were significantly decreased after being seeded in the decellularized IVD (72-fold and 2349-fold, respectively, *P* < 0.05). However, the ratios of collagen type II to type I and type II to III were found to be significantly greater in the decellularized IVD group (9-fold and 16-fold, respectively, *P* < 0.05). Additionally, FOXF1 expression tended to increase in the decellularized IVD group compared with the control group (3-fold, *P* < 0.05). Collectively, these data suggested that decellularized IVD could prevent IVD degeneration.

## DISCUSSION

Tissue regeneration is common choice for various end-stage diseases, and intervertebral disc degeneration is no exception. Here, this study reported an optimized decellularization method that could successfully obtain the IVD ECM scaffold without affecting its spatial framework, bioactivity and biomechanics. The decellularized allogeneic IVD induced minimal immunological reaction. By seeding MSCs into the ECM scaffold, we were surprised to find that IVD-like cell differentiation *in vitro*. Furthermore, this study confirmed the therapeutic effect of decellularzied allogeneic IVD on preventing disc degeneration *in vivo*.

IVD is a bulky and denser tissue. We applied physical treatments (snap freezing-thawing) before the chemical treatments (Triton X-100 and SDS) to disrupt cell membranes and release cellular components. To loosen the IVD tissue and thoroughly expose the cellular antigens to the decellularization reagents, a relatively long time and treatment with low concentration of decellularized reagents (2% Triton X-100 and 1% SDS) has been proven effective. Our results showed that the main bioactivity components of IVD ECM (GAGs, AGN, collagen type II and collagen type I) were well preserved. It should be noted that the content of collagen and water have been ‘increased’ in the optimized decellularization protocol group. It is most likely because decellularization detergents could partially break up peptide bonds and expose more amino and carboxylic groups to form hydrogen bonding with water, which results in higher HYP and water content. A similar paradoxical phenomenon was also observed in other studies [23, 24]. Because insufficient removal of DNA fragments was associated with a greater risk of a pro-inflammatory or immune response and also had potential biosecurity problems [25, 26], we used DNAase to minimize the DNA content (less than 5%). We well-controlled washing condition at a low temperature (4°C) throughout the whole decellularization to reduce ECM ultrastructure damage (caused by proteases releasing from disrupted cells). And the SEM and TEM observations confirmed the good integration and continuity of our ECM microstructure. Besides, no significant decrease of mechanical properties (elastic and compressive modulus) after decellularization was further supporting the above finding. Therefore, based on the combined effect of the aforementioned decellularization procedures, we proposed an optimized decellularization protocol for rabbit intervertebral disc in this study.

For completely removing the cellular components and DNA fragments, we found the decellularized allogenic IVD induced minimal immunological reaction *in vivo*. The histological evaluation showed no obvious host inflammatory cells infiltration and classic foreign body granulomatous inflammation were found in the decellularized allogenic IVD. The degree of cell-mediated immunoreaction (macrophages and CD8+ T-cells) was kept at a low level in the decellularized IVD treated group. And some researchers believed the presence of residual cell remnants, including cellular antigens and DNA fragments, had negative impact on the newly seeded cells [27-29]. Thus, allogenic IVD using the optimal decellularzation method could provide scaffolds with little immunological reaction.

By seeding MSCs into the ECM scaffold, we were surprised to find that IVD-like cell differentiation was induced during the culture process *via* morphological observations and IVD-related genes analysis. The dynamic cell morphology changes (thin and flat to column and strip-like shape) from day 3 to day 21 indicated that the differentiation had occurred in the seeded cells. And we deduced that decellularzied ECM may induced IVD-like cell differentiation *in vitro* for the cell shape was similar to normal IVD cells. It is well confirmed by our IVD-related gene expression results. The significant increases of classic IVD cell marker genes (collagen type II, AGN and SOX-9) and novel marker gene (GPC3) demonstrated that the differentiation of MSCs happened in the ECM is discogenic. For example, the expression of collagen type II (12-fold), AGN (8-fold) and SOX-9 (14-fold) were important for IVD cells homeostasis and usually applied to define the IVD phenotypes. The enhanced collagen type II to type I ratio (8-fold) indicates a discogenic trend in seeded cells rather than fibrogenic. In addition to that, recent studies have regarded GPC3 as a novel candidate IVD marker for distinguishing disc cells from articular chondrocytes [30, 31]. While the mechanism of IVD-like cell differentiation is not fully understood, we suspected that the decellularized ECM may play an important role on it. The well-preserved bioactivity proteins (collagen type II, AGN, collagen type I, and GAGs) and intrinsic microstructures could possible provide the protein ‘footprints’ of the previous resident cells (normal intervertebral disc cells) to guide cell proliferation and differentiation. TGF-β families (secreted factors) and their receptors were associated with MSCs differentiating to IVD cells and had therapeutic effect on disc degeneration by increasing collagen type II and AGN [12, 32-34]. Their high expressions during the latter stages may also had related to the cell differentiation.

The IVD-cell differentiation mechanism was needed to be explored in the following study but we confirmed the therapeutic effect of decellularzied allogeneic IVD on preventing disc degeneration *in vivo*. In the field of disc degeneration, growth factor supplementation and MSC-based therapies have been investigated by many researchers [23, 35-37]. Transplanting MSCs into collagen sponge, actelocollagen and hyaluronan gel to repair disc degeneration (improving the ECM, water content and disc height index (DHI)) has been proven to be effective [38-40]. Recently, Borde B et al and Grunert P et al proposed that high-density collagen is capable of repairing AF defects (induced by needle puncture) [41, 42]. However, there are few studies that have focused on the effect of decellularzied IVD ECM on treating disc degeneration. The outcomes (MRI and histology) of our animal degeneration experiment demonstrated that the injection of decellularized allogeneic ECM microparticles into degenerated disc can improve the degeneration and delay the degeneration process by increasing the water content, disc height, and ECM integrity and content. The regenerating effect of ECM microparticles could be attributed to the native microenvironments, and the active ingredients of ECM were preserved after decellularization. The role of decellularzied allogeneic IVD in preventing disc degeneration *in vivo* may be consistent with the effect on promoting MSC proliferation and differentiation and improving the HDCs phenotypes. Additionally, upon decellularization, the peptide bonds were partially broken up and exposed more amino and carboxylic groups to form hydrogen bonding with water; the resulting ECM scaffold has favorable hydrophilic properties and higher water content in the degenerating disc. Moreover, according to previous research results, high-density decellularized ECM microparticles (which mainly contain collagens and GAGs) could have a positive effect on the AF defect repairs in the injured intervertebral discs [41, 42].

There were several limitations in the present study. Although differentiating MSCs to IVD-like cells using decellularized scaffolds has been successful, other possible approaches are worthy of being pursued. First, different culture conditions, including hypoxia, non-contact co-culture or contact co-culture with IVD cells and supplementation of growth factors, should be further explored. Second, because of the lack of definite IVD cell markers, the specific markers are needed to confirm the differentiation of MSC toward IVD-like cells. Future research directions will focus on establishing the orthotopic whole IVD implantation model and using proteomic approaches to analyse the composition of IVD ECM after decellularization.

## CONCLUSIONS

The current study showed an optimized decellularization method to completely remove the cellular components of the IVD while maintaining a three-dimensional architecture, biochemical characteristics and biomechanical properties. Good immune tolerance and no cytotoxicity of our ECM scaffold were demonstrated. Interestingly, MSCs cultured in the decellularized IVD ECM scaffold were observed to differentiate to IVD-like cells. Moreover, the effects of decellularized IVD allogeneic ECM microparticles on treating disc degeneration and delaying the process of degeneration were revealed. Thus, this study showed that the allogeneic decellularized IVD ECM scaffold has therapeutic potential for treating disc degeneration.

## MATERIALS AND METHODS

### Research overview

The research framework was depicted in Supplemental Figure 1. IVD samples were harvested from rabbits. The cellular components were completely removed but the structure of ECM was well preserved. The determination of an ideal decellularization protocol was based on histological, DNA and collagen content analyses. Additionally, the mechanical properties of decellularized IVD were evaluated by testing the elastic and compressive modulus. To assess the biological safety of allogenic decellularized IVD, it was implanted into allogeneic subcutaneous tissue *in vivo*. MSCs were seeded in decellularized IVD, and the effect of ECM scaffold on MSCs proliferation and differentiation was evaluated using histological, biochemical, and real-time PCR (RT-PCR) methods and SEM. Moreover, decellularized IVD-derived allogenic ECM microparticles were injected *in vivo* to investigate the effect on preventing disc degeneration. Besides, HDCs were also isolated and reseeded into decellularized IVD to observe the effect of decellularized IVD on HDCs degeneration *in vitro*.

### Harvesting of rabbit IVD

IVD samples were collected from the thoracic and lumbar segments of New Zealand White rabbits that were aged approximately four months old (*n* = 55, of which 45 were used for the *in vitro* study and ten for *in vivo* research). Muscle tissues and bone chips were carefully removed from each sample. The cartilage endplates were resected, then lavaged the IVD (including AF and NP) with phosphate-buffered saline (PBS) to eliminate the excess blood. The harvested IVDs were immediately frozen in liquid nitrogen for further use. IVD for DNA and HYP analysis must be freeze-dried *via* ice sublimation under a high vacuum in a vacuum freeze dryer (Marin Christ, Osterode, Germany) to normalize the specimens.

### Decellularization of IVD

An optimized decellularized protocol was necessary to completely remove the cellular components of IVD and maximally retained the complicated three-dimensional structure and biomechanics of the native ECM. To facilitate the diffusion of the cellular content and decellularized reagents, snap freezing-thawing cycles were used prior. Harvested IVDs were placed in a 37°C water bath and liquid nitrogen in rapidly alternating treatments for five cycles. First, 2% Triton X-100 (Sigma) in deionized water was applied to soak the IVDs for 24 h on an orbital shaker at 100 rpm and at 4°C. Second, 1% SDS (Sigma) in deionized water was applied for 24 h to further remove the nuclear remnants and cytoplasmic proteins under the above conditions. The complete removal of DNA remnants from the tissue was fairly difficult due to their ‘sticky’ nature, which caused them to easily adhere to ECM proteins. To minimize the DNA content in the ECM, 200 U/ml DNAase (Sigma) in PBS was applied with shaking at 100 rpm and at 37°C for 12 h. Finally, the IVDs were rinsed with PBS for 6 h to remove the residue reagents effectively. Before establishing the above optimized decellularization protocol, we explored a suboptimal preparative method in which the concentrations of Triton X-100 and SDS in deionized water were 3% and 2%, respectively, and no DNAase was incorporated. By applying the optimized decellularization protocol to IVD, we found that decreasing appropriately the concentration of decellularized agents (Triton X-100 and SDS) will not affect the removal rate of cells and can obvious reduce the damage of ECM. Besides, DNAase can minimize the DNA content and lower risk of a pro-inflammatory or immune response.

### Isolation and culture of rabbit MSCs

Rabbit MSCs were isolated from the bone marrow of four-month-old New Zealand White rabbits (*n* = 5) by gradient isolation of mononuclear cells, and they were cultured in Dulbecco's modified Eagle medium with high glucose (DMEM-HG) (Gibco) plus 10% fetal bovine serum (FBS) (Gibco), 100 U/mL penicillin, 100 μg/mL streptomycin, and 2.5 μg/mL amphotericin B at 37°C in a humidified atmosphere that contained 5% CO_2_. Cells were plated in T-75 flasks and were allowed to adhere for 24 h. Non-adherent cells were removed by three washes with PBS, and the adherent cells were cultured in complete DMEM-HG. Cells were detached using 0.25% trypsin-EDTA upon 80-90% confluence and were subcultured as passage one (P1). Cells at P3 were used for the recellularization of the decellularized IVD.

### Isolation and culture of HDCs

HDCs were collected from five patients with IDD caused by lumbar disc herniation, who underwent discectomy and fusion in the Department of Orthopaedics, Sir Run Run Shaw Affiliated Hospital of Zhejiang University School of Medicine. Informed consent was obtained from each patient, and the research protocol was approved by the Ethics Committee of the Sir Run Run Shaw Affiliated Hospital of Zhejiang University School of Medicine. Bone chips and excess blood were carefully removed from each sample using surgical blades and forceps. The human IVDs were dissected into small pieces using a scissor. The tissues were then digested using 0.2% collagenase solution (Sigma) in standard DMEM for 8 h at 37°C with vibration. After digestion, the debris was filtered out with a cell strainer, and the cells were centrifuged at 800 rpm for 5 min. The HDCs were then cultured in T-75 culture flasks at 37°C in a 5% CO_2_ atmosphere. The cells were also detached using 0.25% trypsin-EDTA upon 80-90% confluence, and they were subcultured as passage P1. The cells at P2 were used for implanting into the decellularized IVD.

### Seeding MSCs into the IVD ECM scaffold

To test the effect of decellularized IVD on MSCs proliferation and differentiation, the ECM samples were incubated in DMEM-HG medium for 12 h at 37°C. A few studies proposed that seeding the cells several times was superior to seeding a single time for the cell viability and distribution in ECM [27, 44]. In this study, a concentration of approximately 7×10^6^ cells/mL rabbit MSCs were introduced at each step, for a total of three steps, with 1 h intervals between each step. After 1 h of finishing the third seeding to allow sufficient adhesion to the scaffold, DMEM-HG medium was then carefully supplemented to a 24-well plate that contained recellularized scaffold (Supplemental Figure 1 and Supplemental Figure 2). The same source of MSCs was seeded in a Petri dish with a concentration of 5×10^5^ cell/mL and used as the negative group.

### Seeding HDCs into the IVD ECM scaffold

It is believed that ECM can delay replicative senescent chondrocyte (which is similar to an IDD cell) dedifferentiation and enhance redifferentiation [45]. We therefore sought to seed 7×10^6^ cell/mL HDCs into decellularized IVD to evaluate the improved effect on degenerative cells. Cells from the same source were seeded in a Petri dish at a concentration of 5×10^5^ cell/mL and used as the corresponding negative control group.

### Rabbit Subcutaneous implantation of decellularized IVD

For allograft cellular components can lead to immune reaction and affect the treatment effect, so allograft transplant was used to test the immunogenicity of decellularized IVD. New Zealand White rabbits (*n* = 5) were utilized for the study. Each rabbit was anesthetized with an intraperitoneal injection of chloral hydrate (5 ml/kg). Three subdermal pockets were made on either side of the dorsal midline of each rabbit by making small incisions (1 cm). Decellularized IVDs (*n* = 3) and normal IVDs (*n* = 3) were separately implanted into corresponding pockets through the incision. Incisions were closed with surgical ligation. One month after implantation, implants were removed and prepared for histological evaluation by submersion in 4% PBS-buffered paraformaldehyde (PFA).

### Evaluation of histological, histochemical, immunohistochemical and fluorescent staining

The normal IVD, decellularized IVD, subcutis-embedded specimens and recellularized decellularization IVD at 3, 7, 14 and 21 days were washed three times with PBS and fixed in 4% PFA for 24 h, followed by standard paraffin embedding and sectioning. For treatment of IDD study, IVD samples were harvested from rabbits at 2 months after injection of decellularized IVD-derived ECM microparticles, fixed in 4% PFA for 48 h and decalcified for at least 30 days before embedding in paraffin wax. After sectioning, routine H&E staining was performed to reveal the complete distribution of various cells and cells with different morphologies. Alcian blue staining was used to identify the GAG content that was retained in decellularized IVD, recellularized decellularization IVD and IVD samples. Specimens for immunohistochemistry were possessed as previously described by our group [46]. Epitopes of interest included collagen type II (Novus Biologicals, Littleton, USA), AGN (Novus Biologicals, Littleton, USA), collagen type I (Abcam, Cambridge, US), CD8 (Novus Biologicals, Littleton, USA) and MAC387 (Abcam, Cambridge, US). The hematoxylin and Vectastain ABC kit (Vector Laboratories, Burlingame, USA) was used for visualization of the cell nuclei and the positive signal in the specimens, respectively. Specimens that were incubated for 5 min in DAPI (Sigma) fluorescent nuclei stain were used to observe the proliferation of the seeded cells in decellularized IVD at different time points.

### Determination of DNA, collagen and water content

The content of total genomic DNA of normal IVD, decellularized IVD, and recellularized decellularization IVD at different time points was extracted and isolated using a DNeasy Blood & Tissue Kit (Qiagen, Hilden, Germany) in accordance with the manufacturer's protocol and was previously described by our group [47]. The DNA content was measured with a NanoDrop 8000 (Thermo Fisher Scientific, Wilmington, USA). As a marker amino acid of collagen, the content of HYP can be measured using a spectrophotometric method [48]. The amount of HYP in the samples was determined by a calibration curve with a linear region between 0.2 and 1 mg/100 mL, prepared from HYP standards (Sigma). The mass changes of normal and decellularized IVDs after freeze-drying were calculated to evaluate the water content.

### Scanning and transmission electron microscopy

SEM and TEM were performed to examine the micro-architecture of the normal and decellularized IVD. NP and AF tissue samples were dissected out and fixed in 2.5% glutaraldehyde in PBS overnight at 4°C, followed by three washes in PBS. The fixed samples were post-fixed with 1% OsO4 for 1 h and washed with PBS three times, as above. The samples were then dehydrated in a graded series of ethanol and dried in a critical point dryer (HCP-2, Hitachi, Japan) with liquid CO_2_. Subsequently, the dried samples were sputter-coated with gold-palladium and viewed under the SEM (EM-3200, KYKY, China).

For the TEM, we fixed and post-fixed AF and NP samples as above. The samples were infiltrated in a 1:1 mixture of absolute acetone and resin for 1 h, and then, they were transferred to a 1:3 mixture of absolute acetone and the final resin for 3 h and, then, transferred to 100% resin overnight. The samples were placed in capsules that contained embedding medium and were heated at 70°C for 9 h. The hardened blocks were ultra-thin sectioned at 70 nm with a diamond knife using an ultramicrotome (EM UC7, Leica, Germany) and placed on copper grids. Then, the specimen sections were stained by uranyl acetate and alkaline lead citrate for 15 min and observed by a TEM (H-7650, Hitachi, Japan).

### Cytocompatibility testing of decellularized IVD

The decellularized IVDs were incubated in DMEM-HG medium for 48 h in a humidified atmosphere with 5% CO_2_ at 37°C, and the medium was collected for later use (leaching solution). The leaching solution was generated by each decellularized IVD incubated in the 1 ml DMEM-HG medium. MSCs at P2 were seeded in 96-well cell culture plates in 200 μl standard DMEM-HG medium at a concentration of 5×10^3^ cells/well for 24 h. The medium was then removed and replaced with the leaching solution (25%, 50% and 100%) and incubated for six days. CCK-8 was added to each well of the plate, and the plate was incubated for 3 h in the incubator at the time points of one, two, three, four, five and six days. The Live-Dead cell staining (BioVision, Milpitas, USA) was used to distinguish between live and dead cells and observe the overview of the situation of cellular activity. The detailed description of CCK-8 and Live-Dead cell staining process can be obtained in our previous studies [47, 49]. The stained live (green) MSCs at the above time points can be visualized by fluorescence microscopy. The metabolic activity of MSCs was detected by CCK-8 and compared with the positive group (cells cultured in standard DMEM-HG medium) to determine the cytocompatibility of the decellularized IVD samples.

### Biomechanical testing

The mechanical properties of AF and NP after decellularization were measured using uniaxial tensile and dynamic strain tests, which performed using a computer-controlled test machine (Z2.5, Zwick/Roell, Germany) and had been described and validated by our previous studies [50-52]. For AF, the samples were clamped to the grips in the mechanical apparatus, and the initial specimen's width, length and thickness were recorded. The samples were then stretched to tensile failure at a rate of 10 mm/min. The stress-strain curves were collected. The UTS was calculated by dividing the maximum load by the cross-sectional area of the specimen. The maximum elongation was calculated by dividing the strain at a failure length by the initial length of the specimen. The elastic modulus was calculated from the slope of the ascending linear region of the stress-strain curve. For NP, the samples were maintained in 37°C PBS until testing. The samples were mounted into the specific round hole (4.6 mm in diameter and 3 mm deep), and the top loading shaft was then slowly loaded to the NP. The oscillatory amplitude was set at 0.3 mm, which generated a dynamic strain level of 10%. The dynamic strain was applied at the loading frequencies of 1 Hz, which was similar to physiological conditions. The compressive modulus of NP at 1 Hz was calculated after the whole test cycle from the slope of the ascending linear region of the stress-strain curve.

### Cell viability and metabolic activity of the recellularized decellularization IVD

The viability of cells in the recellularized decellularization IVD was determined by Live-Dead cell staining. The samples were incubated with calcein acetoxymethyl ester and ethidiumhomodimer-1 for 20 min at 37°C in darkness, to simultaneously but separately stain both the living and dead cells. After washing with PBS to remove the labeling reagents, the stained samples were visualized by fluorescence microscopy. The metabolic activity of the recellularized decellularization IVD cells was assessed by CCK-8, and the incubating time was adjusted to be 2 h. The corresponding absorbance was transformed into cell numbers by using the standard curve.

### 5.15. Gene expression of IVD cell markers by RT-PCR

Because the ECM proteins which newly deposited by recellularized decellularization IVD cells was difficult to distinguish from the ECM that remained after decellularization, gene expression rather than protein expression of a panel of IVD cell markers was evaluated. RNA from MSCs-seeded decellularized IVD, HDCs-seeded decellularized IVD and monolayer controls was isolated using RNeasy Mini kit (Qiagen, Valencia, CA, USA) according to the instructions [49]. RNA integrity and quantification was detected by using NanoDrop 8000. It was then reverse transcribed using 1 μg of RNA from each sample, 2 μl of 5×PrimeScript RT Master Mix (Takara Bio, Otsu, Japan), and 4 μl of RNase Free dH2O in a total volume of 10 μL. After amplifying the cDNA, RT-PCR was performed on a 96-well plate ABI Prism 7500 (Applied Biosystems, Foster City, CA, USA) using SsoFast EvaGreen supermix (Bio-Rad, Hercules, CA, USA). The total volume (20 μL) of each PCR reaction contained 10 μL of SsoFast EvaGreen supermix, 7 μL ddH2O, 2 μL cDNA and 10 μM of each of the forward and reverse primers (Supplemental table 1). The expression levels of thirteen genes (collagen type II, collagen type I, SOX-9, GPC3, FOXF1, AGN, CA12, TIMP-1, TIMP-2, TGF-β families and their receptors) were chosen to investigate the progress of IVD regeneration and its possible mechanism. In addition, the expression levels of collagen type II, collagen type I, collagen type III and FOFX1 in HDCs seeded decellularized IVDs were used to evaluate whether decellularized IVD has effect on degenerative cells. The expression ratio for each marker was quantified mathematically by the 2^−ΔΔCt^ method using β-actin or 18S rRNA as the housekeeping gene, and the target genes were compared relative to the cells cultured in standard DMEM-HG medium.

### Establishment of the rabbit IVD degeneration model

IVD degeneration was induced by the validated rabbit puncture model [53]. This puncture model initiated a reliable cascade of MRI and histologic changes that resembled the hallmarks of human IVD degeneration (darkening and collapse of disc spaces). Prior to the procedure, the animals were put under general anesthesia. The rabbit's spine was exposed from an anterolateral retroperitoneal approach with aseptic techniques. L4-L5 and L3-L4 IVDs were sequentially punctured with a 22-gauge needle to a depth of 5 mm, and then, the surgical incisions were closed. L5-L6 was left uninjured as the normal control. The rabbits were allowed to move freely around their cages.

### Injection of decellularized IVD-derived ECM microparticles

After cutting the decellularized IVD samples into pieces, an appropriate amount of sterile saline was adding, and then, tissue homogenizer (Bertin * Precellys 24, Bertin Technologies) was applied to grind the samples at 6000 rpm for 30 seconds for approximately 20 circles. The temperature during homogenization should remain at 4°C to prevent protein denaturation. After homogenization, the suspension was obtained and screened through the 80-mesh sample sieve (0.2 mm). The size of the decellularized IVD-derived ECM microparticles (suspension of homogenised ECM) was controlled to be less than 200 μm. The concentration of the DID-derived ECM microparticles suspension was 50 mg/ml.

Injection of the decellularized IVD-derived ECM microparticles was performed one month after degeneration induction. L5-L6 was used as the control group. L4-L5 and L3-L4 were assigned to two groups, namely, the decellularized IVD group and saline group, respectively. Under general anaesthesia and guided by X-rays, L5-L6, L4-L5, and L3-L4 IVDs were exposed from the other side (contralateral to the previous puncture surgery). L4-L5 and L3-L4 IVDs were injected with a 50 μl suspension of ECM microparticles and a 50 μl sterile saline through a Hamilton syringe with a 27-gauge needle, respectively. The small needle size was selected to avoid exacerbating further degeneration.

### MRI evaluation

MRI was performed with a 1.5-Tsystem (GE Signa Excite; GE Healthcare, Milwaukee, Wisconsin) on the rabbits at 0 month, 1 month, 2 months and 3 months to indicate the water content and heights of the discs. Rabbits were placed supine, and the human lumbar sequences with a knee-joint surface coil were used. First, T2-weighted coronal scouting images were acquired at the lumbar spine, and the image with the most intervertebral discs was selected. Then, T2-weighted sagittal scouting images were acquired according to the previously selected coronal locating image. Additionally, a central sagittal image of the lumbar spine was selected as a locating image for the next cross-sectional scans. The middle images for each of the IVD locations at L5-L6, L4-L5, and L3-L4 were selected by locating lines on sagittal plane MRI scans. The window width and window level were 3600 and 1800 for fat-suppressed T2-weighted images, and detailed manipulation were previously described by our group [54].

Measurements were made with the software of ImageJ 1.46 (National Institutes of Health, http://rsbweb.nih.gov/ij/download.html). The mean signal intensity values of T2-weighted imaging of IVD and cerebrospinal fluid in the central sagittal plane were measured, and the ratio between them was calculated to reflect the water content of each IVD. The height of the IVD was also measured and was expressed as the DHI. The average measurement of the anterior, middle and posterior height of the disc was divided by the standard calibration line of each image. The change in the DHI was expressed as the percentage DHI (%DHI) and normalized to the DHI that was obtained from the pre-degeneration (0 month) T2-weighted imaging. The above measurements were performed by 3 people (2 radiologists and 1 spine surgeon) independently, and the results were averaged.

### Statistical analysis

Quantitative results are represented as a mean ± standard deviation. One-way ANOVA or two-way ANOVA was performed to detect the differences in the content of DNA, HYP and water, the mechanical properties, expression levels of the genes, IVD functional outcomes and cytocompatibility among different treatment groups. Statistical analysis was performed using SPSS 19.0 software (SPSS Inc, Chicago, USA), and significance was defined as *P* < 0.05.

## SUPPLEMENTARY MATERIAL TABLE AND FIGURES


