# Supplementary material for: Decellularized allogeneic intervertebral disc: natural biomaterials for regenerating disc degeneration
Source: Oncotarget. 2016 Feb 25;7(11):12121–36. doi: 10.18632/oncotarget.7735 (PMC4914273; doi:10.18632/oncotarget.7735)
Supplement: Supplementary file 1 [file oncotarget-07-12121-s001.pdf]

Decellularized allogeneic intervertebral disc: natural biomaterials for regenerating disc degeneration

Supplementary Material

Supplemental table 1: Rabbit and Human primer sequences used for RT-PCR analysis.

Gene name (Abbr.)	Species	Forward primer sequences	Reverse primer sequences
Collagen type II	Rabbit	GTGGTGACAAAGGCGAAAAG	CCTTCTCGTCAAATCCTCCA
Collagen type I	Rabbit	AGAACGGAGATGACGGAGAAG	CACCATCCAAACCACTGAAAC
SOX-9	Rabbit	CGAACGCACATCAAGACG	AAGGTGGAGTAGAGGCTGGA
Glypican 3 (GPC3)	Rabbit	TGCCCATTCTCAACAACG	TCCCTTCTTCGGCTGGAT
Forkhead box F1 (FOXF1)	Rabbit	CCGCAAGGAGTTCGTGTT	CACGGCTTGATGTCTTGG
Aggrecan (AGN)	Rabbit	GGAGTTCTTTTTGGGAGTGGT	CAGGTCAGGGATTCTGTGTGT
Arbonic anhydrase 12 (CA-12)	Rabbit	CTGGCGTTCTTGGCATCT	GCCTCGGTCTCCATCTTGA

Tissue inhibitor of metalloproteinase 1 (TIMP-1)	Rabbit	CAACTCCGACCTTGTCATCAG	TACCCGCAGACACTTTCCAT
Tissue inhibitor of metalloproteinase 2 (TIMP-2)	Rabbit	GTGGGAGGCAAGAAGGAGTAT	TTGATGTTCTTCTCCGTGACC
Transforming growth factor β2 (TGFβ2)	Rabbit	GGGCAGATCCTAAGCAAGC	CCTGGTGCTGTTGTAGATGG
Transforming growth factor β3 (TGFβ3)	Rabbit	CCAATTACTGCTTCCGCAAC	TAGTAGCCCTTGGGTTCGTG
Transforming growth factor β1 (TGFβ1) receptor	Rabbit	GTTCGGGATCAGGTTTACCA	TCGCCAAACTTCTCCAAATC
Transforming growth factor β2 (TGFβ2) receptor	Rabbit	CCCATCGTGCATAGAGACCT	ACAGACAGCGTAGGGTCCAG
β-actin	Rabbit	AACTAAGAACGGCCATGCAC	AACTAAGAACGGCCATGCAC
Collagen type II	Human	CTGGAAAAGCTGGTGAAAGG	GGCCTGGATAACCTCTGTGA
Collagen type I	Human	GGCCCAGAAGAACTGGTACA	AATCCATCGGTCATGCTCTC
Collagen type III	Human	TCAGGGTGTCAAGGGTGAA	CAGGGTTTCCATCTCTTCCA
FOXF1	Human	TGTGACCGAAAGGAGTTTGTC	GGCTTGATGTCTTGGTAGGTG
18S rRNA	Human	CCTGCGGCTTAATTTGACTC	AACTAAGAACGGCCATGCAC

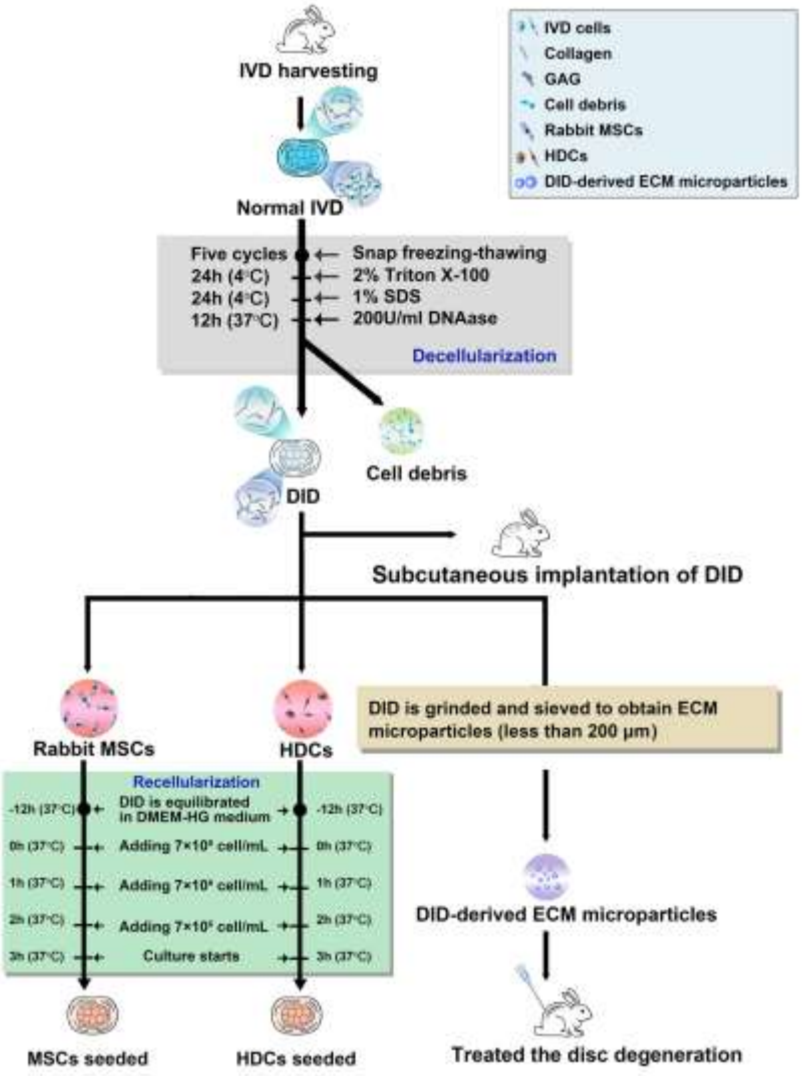
IVD harvesting
IVD cells
Collagen
GAG
Cell debris
Rabbit MSCs
HDCs
DID-derived ECM microparticles
Normal IVD
Five cycles
Snap freezing-thawing
24h (4°C)
2% Triton X-100
24h (4°C)
1% SDS
12h (37°C)
200U/ml DNAase
Decellularization
Cell debris
DID
Subcutaneous implantation of DID
Rabbit MSCs
HDCs
DID is grinded and sieved to obtain ECM microparticles (less than 200 μm)
Recellularization
-12h (37°C)
DID is equilibrated in DMEM-HG medium
0h (37°C)
1h (37°C)
2h (37°C)
3h (37°C)
Culture starts
DID-derived ECM microparticles
MSCs seeded
HDCs seeded
Treated the disc degeneration

Supplemental figure 1: Schematic representation of the overall research. Abbreviations: IVD = intervertebral disc, DID = decellularized IVD, MSCs = mesenchymal stem cells, HDCs = human-derived IVD degeneration cells, GAG = glycosaminoglycan.

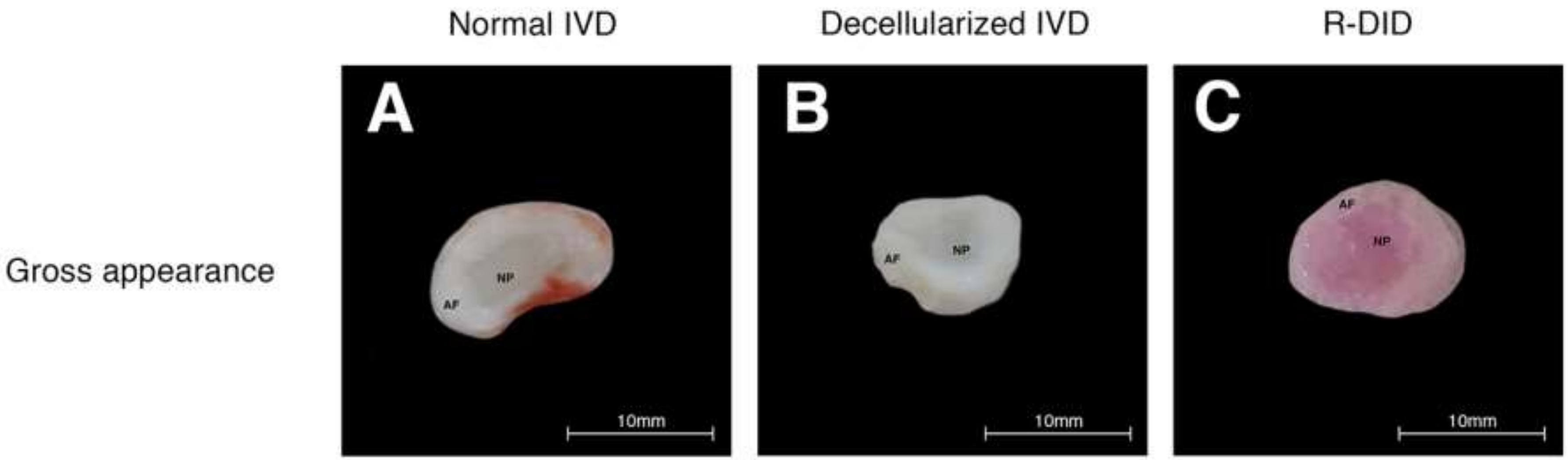

Supplemental figure 2: Macroscopic images of normal IVD **A.** decellularized IVD **B.** and recellularized decellularization IVD **C.** Abbreviations: IVD = intervertebral disc, R-DID = MSCs seeded decellularzied IVD ECM.

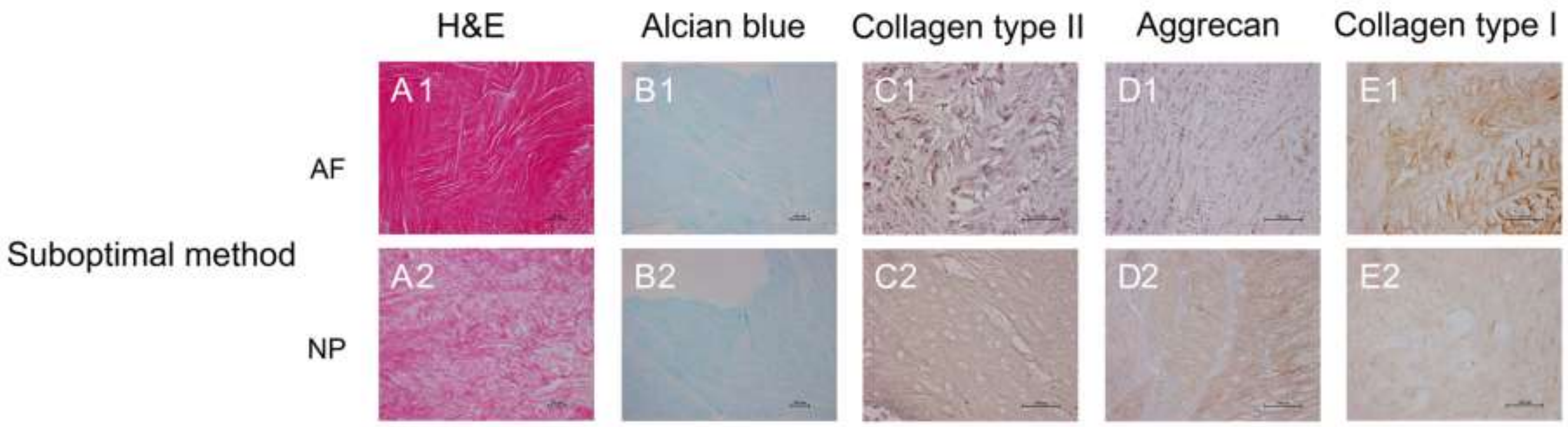

Supplemental figure 3: Histological examination of suboptimal method. Suboptimal method includes 3% Triton X-100 for 24 h and 2% SDS for 24 h. Abbreviations: AF = annulus fibrous, NP = nucleus pulposus.

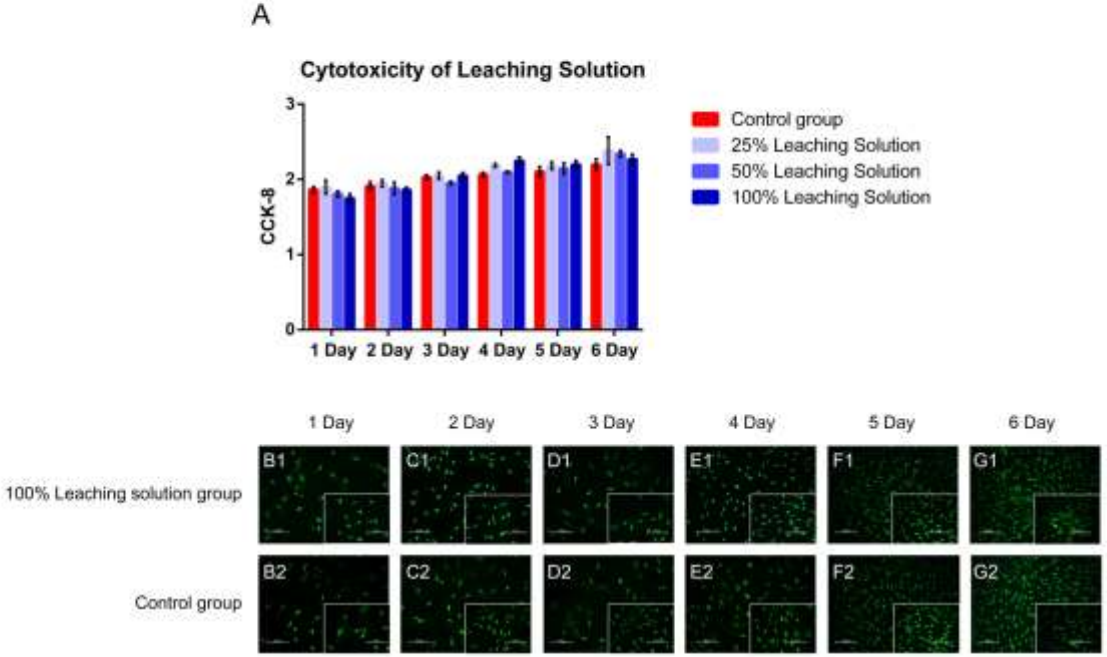

Supplemental figure 4: Confirming the biocompatibility of ECM scaffolds *in vitro*. The cytocompatibility of different decellularized IVD leaching solution concentrations was assessed by CCK-8 (A) and Live-Dead cell staining compared with standard DMEM-HG medium (B, C, D, E, F and G).

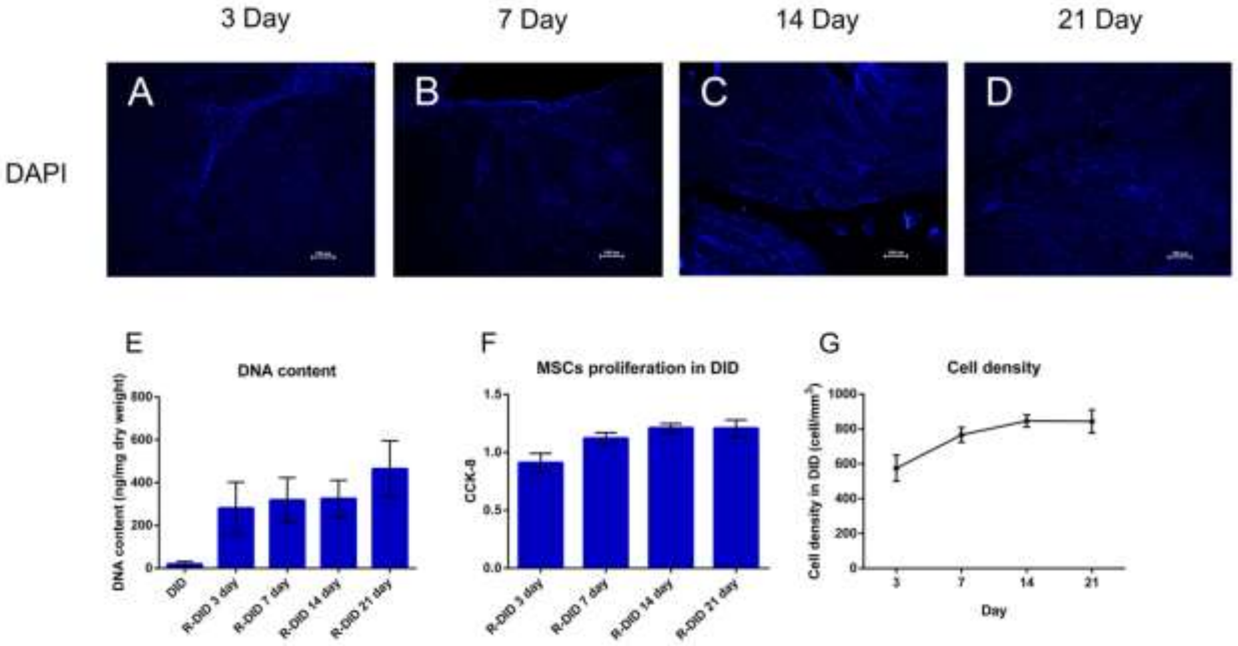

Supplemental figure 5: Decellularized IVD ECM supports MSC proliferation *in vitro.* DAPI (**A-D**) staining, DNA content (**E**) and CCK-8 test (**F** and **G**) showed the MSC proliferation process. Abbreviations: DID = decellularized IVD, R-DID = MSCs seeded decellularzied IVD ECM.

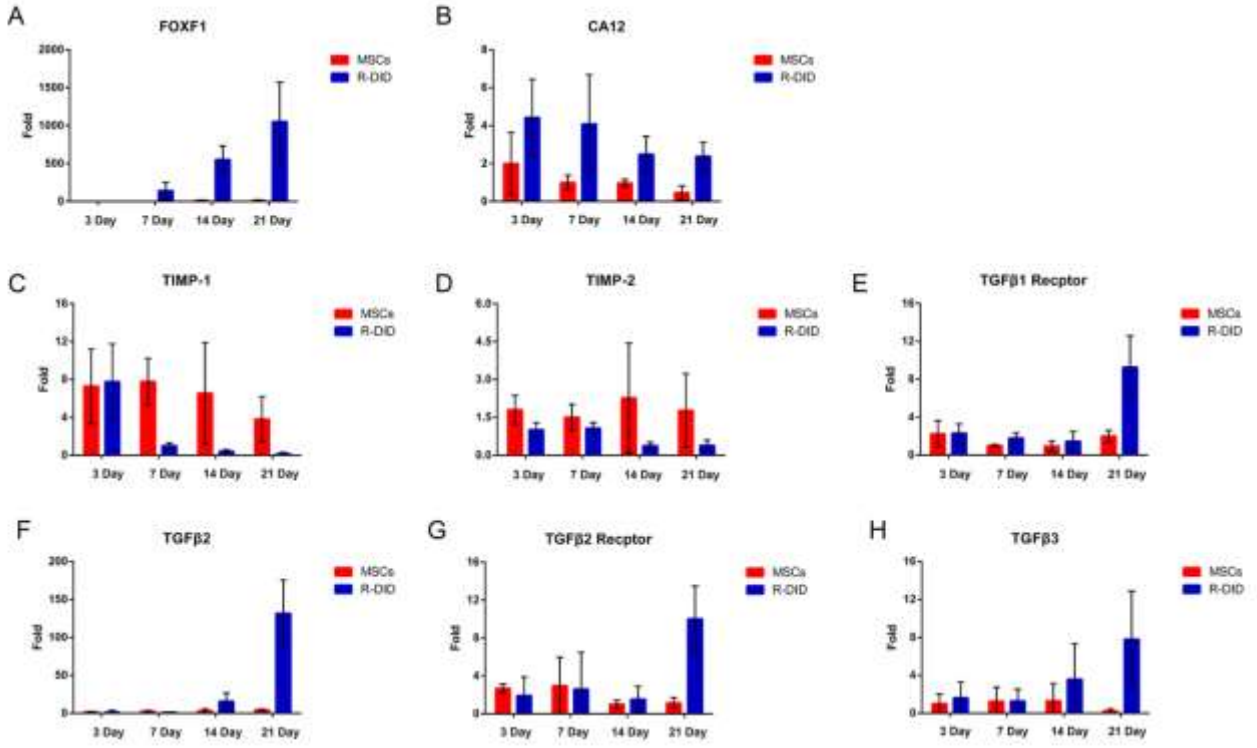

Supplemental figure 6: The IVD-related gene expression of MSC seeded in IVD ECM by RT-PCR. Gene expression data are normalized to the average number. MSCs: Cells cultured in standard DMEM-HG medium (n = 8 per study group per time point). Abbreviations: R-DID = MSCs seeded decellularzied IVD ECM.

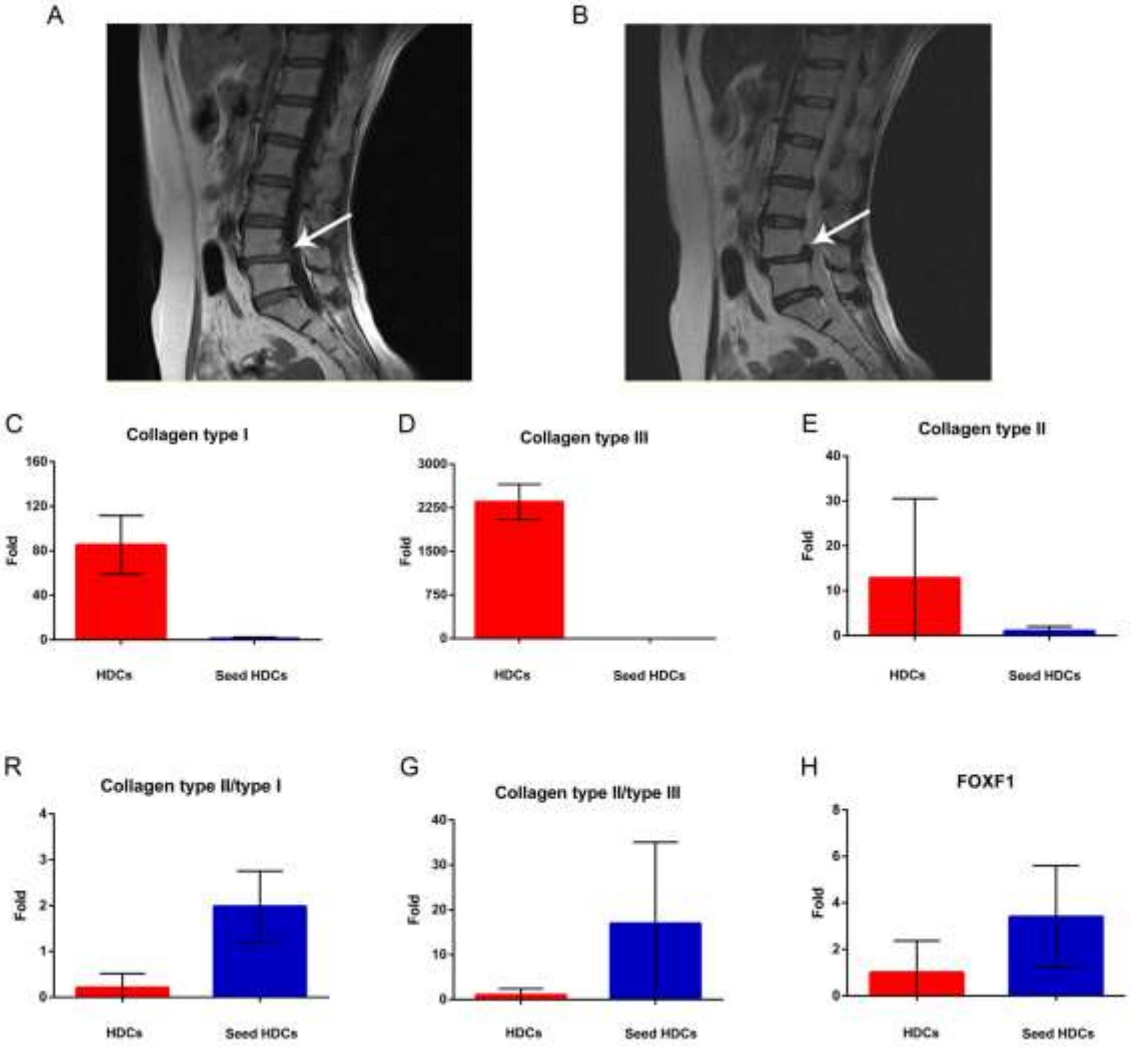
A
B
C
Collagen type I
Fold
HDCs
Seed HDCs
D
Collagen type III
Fold
HDCs
Seed HDCs
E
Collagen type II
Fold
HDCs
Seed HDCs
R
Collagen type II/type I
Fold
HDCs
Seed HDCs
G
Collagen type II/type III
Fold
HDCs
Seed HDCs
H
FOXF1
Fold
HDCs
Seed HDCs

Supplemental figure 7: Representative MRI image of an IDD patient (A and B) and gene expression of the degenerative cells' phenotypes after seeding into decellularized IVD for seven days (C, D, E, F, G and H). Gene expression data are normalized to the average number (n = 5 per study group). Abbreviations: HDCs = human degenerative disc cells cultured in standard DMEM-HG medium, Seed HDCs = HDCs seeded in decellularzied IVD ECM.
